# Relationship of Tooth Wear to Chronological Age among Indigenous Amazon Populations

**DOI:** 10.1371/journal.pone.0116138

**Published:** 2015-01-20

**Authors:** Elma Pinto Vieira, Mayara Silva Barbosa, Cátia Cardoso Abdo Quintão, David Normando

**Affiliations:** 1 Dental School, Department of Orthodontics, Federal University of Pará-UFPA, Faculty of Dentistry, Belém, Brazil; 2 Dental School, Department of Orthodontics, State University of Rio de Janeiro-UERJ, Rio de Janeiro, Brazil; 3 Department of Orthodontics, State University of Rio de Janeiro-UERJ, Rio de Janeiro, Brazil; 4 Department of Orthodontics, Federal University of Pará-UFPA, Faculty of Dentistry, Belém, Brazil; Ohio State University, UNITED STATES

## Abstract

In indigenous populations, age can be estimated based on family structure and physical examination. However, the accuracy of such methods is questionable. The aim of this cross-sectional study was to evaluate occlusal tooth wear related to estimated age in the remote indigenous populations of the Xingu River, Amazon. Two hundred and twenty three semi-isolated indigenous subjects with permanent dentition from the Arara (n = 117), Xicrin-Kayapó (n = 60) and Assurini (n = 46) villages were examined. The control group consisted of 40 non-indigenous individuals living in an urban area in the Amazon basin (Belem). A modified tooth wear index was applied and then associated with chronological age by linear regression analysis. A strong association was found between tooth wear and chronological age in the indigenous populations (p <0.001). Tooth wear measurements were able to explain 86% of the variation in the ages of the Arara sample, 70% of the Xicrin-Kaiapó sample and 65% of the Assurini sample. In the urban control sample, only 12% of ages could be determined by tooth wear. These findings suggest that tooth wear is a poor estimator of chronological age in the urban population; however, it has a strong association with age for the more remote indigenous populations. Consequently, these findings suggest that a simple tooth wear evaluation method, as described and applied in this study, can be used to provide a straightforward and efficient means to assist in age determination of newly contacted indigenous groups.

## Introduction

The dentition is a useful forensic source since it is available long after all other tissues have disintegrated [1–5]. Teeth can provide a reliable marker for the estimation of chronological age before adulthood [1, 6–10], since their development is less affected by malnutrition and other systemic factors than is skeletal development [11]. Measures of dental wear can also provide a means to estimate age, and are especially useful after the dentition has finished developing. However, dental wear is affected not only by the time the dentition has been in use (i.e., age) but also by dietary factors.

The need for a reliable indicator for age estimation, especially in adults, has stimulated several studies aiming to create new techniques to estimate adult chronological age [1, 6–9, 12, 13]. In indigenous populations, age can be estimated based on family structure and physical examination performed by a physician, dentist or anthropologist. These methods are limited because the main criteria applied, skeletal and dental development, cannot be applied to adults. In addition, the reference values frequently used are guided by studies in urban populations.

Occlusal tooth wear is considered a natural physiological process during aging [6, 8, 10, 14–16], with its etiology attributed to the mechanisms of abrasion, erosion and attrition [10, 14, 16]. These mechanisms rarely operate in isolation. The co-occurence of two or more different processes at different times increases the complexity of tooth wear [14, 16, 17]. The amount of tooth structure lost varies significantly amongst different population groups according to eating habits and methods of food preparation [1–3, 6, 18]. Other factors such as the loss of teeth, parafunctional habits, changing patterns of mandibular movement, bite force, geographic and environmental conditions, might also influence wear processes [3, 10, 14, 17, 19, 20]. Studies confirm that degenerative diseases of teeth vary amongst different ethnic groups [6, 9, 11, 14, 21]. For this reason, it is important to perform investigations with different populations in order to evaluate the accuracy of tooth wear as an estimator of chronological age.

## Materials and Methods

### Ethics statement

This study was approved by the National Ethical Committee (CONEP) for Health Sciences of Brazil registered under number 25000.066559/2010–11. For inclusion in this study participants provided their written or verbal informed consent which was translated into their original language. For those adults that could not sign, verbal consent was obtained through audio recording. Informed consent was also obtained from guardians on behalf of the minors/children enrolled in the study. The ethics committee approved the obtaining of consent through written or verbal recording. Furthermore, legal permission for entry into Brazilian indigenous locations was obtained by FUNAI (National Indigenous Foundation). All future permissions for entry into indigenous areas in Brazil must be obtained through this agency. This study conforms to the STROBE guideline.

### Studied samples

The subjects comprised two hundred and twenty three (223) indigenous people from the Middle Valley of Xingu river, Pará, Brazil, of which one hundred and seventeen (117) belonged to the ‘Arara’ group, sixty (60) to the ‘Xicrin- Kaiapó’ and fourty-six (46) to the ‘Assurini’ groups. Assessment was also performed on a control group of forty (40) non-indigenous individuals belonging to the urban population of the city of Belém, the capital of Pará state, Brazil (Table 1). All subjects examined had permanent dentition present with all teeth erupted, except for the third molars. Individuals with eight or more missing teeth had been excluded since a large number of missing teeth can influence the amount of wear on the remaining dentition and tissues. However, this selection process tended to remove the older individuals from the study as they were more likely to have more missing teeth than younger subjects.

**Table 1:** Sample size (n), mean and range of tooth wear and age for the indigenous groups Assurini, Xicrin-Kaiapó and Arara and the control urban population (Belém).

**Group**	**n**	**Tooth Wear Mean (Min-Max)**	**Age (years) Mean (Min-Max)**
**Assurini**	46	0.82 (0.0–2.3)	19.16 (10.8–45.8)
**Xicrin-Kaiapó**	60	0.63 (0.0–2.3)	21.51(10.8–49.3)
**Arara**	117	0.91(0.0–2.9)	21.27 (10.3–48.1)
**Belém city**	40	0.90 (0.2–1.6)	22.25 (13.1–42.4)

Since the relationship between tooth wear and age weakens with increasing age, by removing these individuals the strength of the relationship between tooth wear and age might be overestimated.

Molars were excluded from analysis due to the large number of subjects who had these teeth extracted.

### Tooth wear analysis

**Figure 1:**
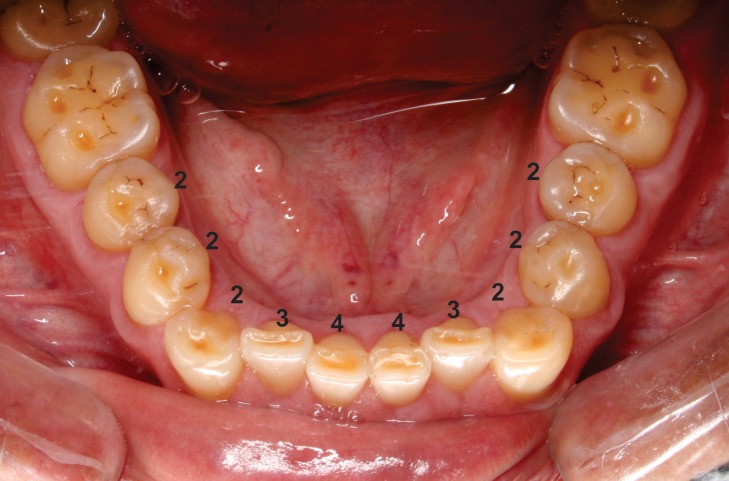
The modified tooth wear examination. The index of occlusal wear for a 48-year- old indigenous person.

Tooth wear was examined in the permanent dentition by the application of a classification system that has been previously described, [22] with minor modification. The occlusal surfaces of the second and first premolars, canines and lateral and central incisors in both dental arches were examined with the aid of good quality illumination. The following scores were recorded for each tooth: 0 = no wear; stage 1 = wear of the enamel only; stage 2 = wear of the dentin, where the occlusal surface had more enamel than dentin; stage 3 = wear of the dentin, where the occlusal surface presents more dentin than enamel; or stage 4 = advanced wear stage, either close to or with exposure of pulp chamber. A previous analysis of the regression coefficient of dental wear for each tooth (I1, I2, C, P1, P2) showed a weak to moderate association with chronological age, so for each individual an arithmetic mean of tooth wear was obtained (Fig. 1).

No significant differences were observed between males and females (*p*> 0.05) from the study sample; these two groups were therefore combined.

Birth records held by nursing assistants responsible for the villages were used to obtain dates of birth of the subjects.

### Statistical analysis

Due to the long distances involved in reaching the villages (around 3–4 days travel from Altamira city by small motorboat), occlusal dental photographs were obtained. Intra-class correlation was used to test the reliability of the tooth wear measurements. The mean scores obtained during the clinical evaluation were compared to those obtained from the occlusal photographs of 20 subjects. The age determination, by tooth wear, was examined statistically by means of linear regression analysis at *p*<0.05, using Minitab software (version 17).

## Results

Tooth wear measurements showed excellent intra-observer reliability (r = 0.78–0.94, *p*<0.0001). However, among the 20 pairs of ratings, the values obtained when examining tooth wear via occlusal photographs were slightly higher in 18 of 20 the re-tested subjects. These findings indicate that this method tends to slightly overestimate the level of tooth wear when compared to a direct clinical examination.

**Figure 2:**
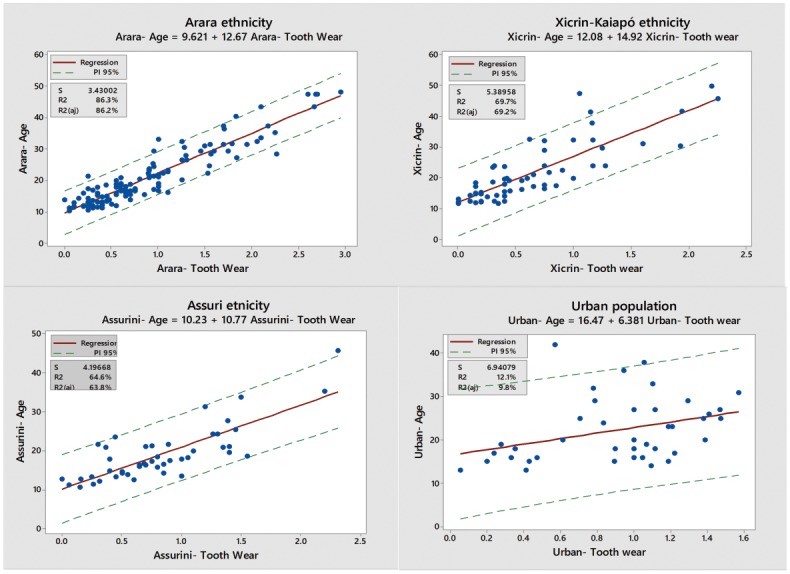
Association between tooth wear and age for the Assurini ethnicity (A), Xicrin-Kaiapó ethnicity (B), Arara ethnicity (C) and urban population- Belém (D).

**Figure 3:**
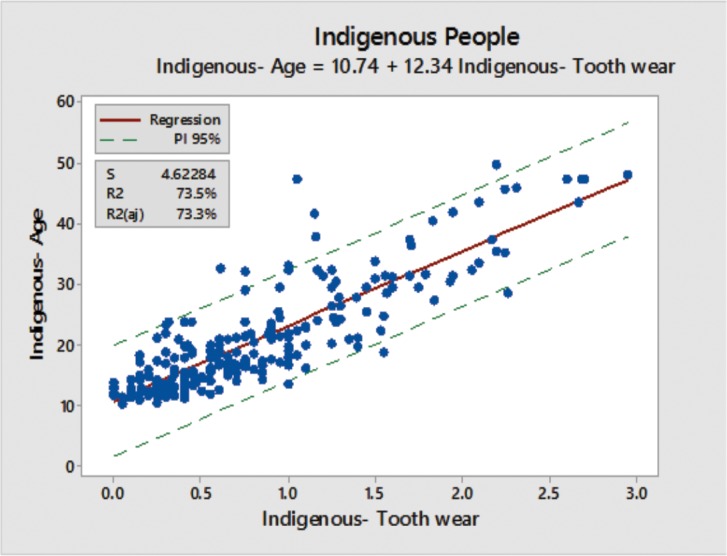
A single model including all 3 populations (Assurini + Xicrin-Kaiapó + Arara).

A strong relationship between tooth wear and chronological age in the three indigenous groups was observed (Figs. 2 and 3). Tooth wear was able to explain 86% of age variability in the ‘Arara’ group (p <0.001), 70% in the ‘Xicrin-Kaiapós’ (p <0.001) and 65% in the ‘Assurini’ groups (p <0.001). It was observed that a 95% prediction interval was higher for the Assurini and Xicrin- Kaiapó groups (Table 2). For the control urban population group, no statistically significant association was found between tooth wear and chronological age (Fig. 2). Only 12% of the age variability could be explained by the degree of tooth wear (R2 = 0.10, p >0.05).

**Table 2:** 95% prediction interval for indigenous and urban population.

**Tooth Wear**	**95% Prediction Interval—Age**			
	**Arara**	**Assurini**	**Xicrin-Kaiapó**	**Urban**
**0.5**	11.2–19.7	8.9–23.9	10.3–29.8	5.7–34.1
**1**	15.8–28.8	12.8–29.6	16.5–37.8	9.1–37.1
**1.5**	22.4–35.2	18.1–35.2	23.6–45.4	11.8–41.3
**2**	28.4–41.8	23.1–41.1	30.6–52.9	--
**2.5**	34.5–48.1	--	--	--

Estimations of age using tooth wear with a 95% prediction interval are presented in Table 2. The data show a small prediction interval for the indigenous people; however, the urban population shows a large interval.

## Discussion

High levels of tooth wear might be considered a response to the effect of abrasive food sources. Increased levels of attrition are founded in populations with a diet based on abrasive and unrefined food when compared to modern, more urban populations [5, 14, 23, 24]. The eating habits of the indigenous tribes that inhabit the Xingu region are very similar across tribal groups, and traditional diets are predominantly based on cassava, nuts, fish, wild meat, sweet potatoes, yams and several fruits [23]. Commercially produced and packaged foods are rarely eaten due to geographic isolation, cost, and lack of regular transport links between villages and urban centers. Teeth are often used as “tools” in traditional communities. Expressions such as “using teeth as tools” and “a third hand” have been used to describe prehistoric reconstructions of human behavior [25].

As reported, some correlation has been observed between chronological age and dental wear especially in ancestral populations [1, 7, 14, 21]. The strong correlation found between age and tooth wear amongst the indigenous people of the Xingu region can be explained by the traditional dietary habits which are still maintained today. While the villages of all three ethnic groups studied were difficult to access, the Arara villages were particularly difficult. Their customs and eating habits are more traditional, which may explain their higher correlation between age and tooth wear.

Currently the diet for urban populations is based on softer, commercially prepared and packaged food which significantly reduces the level of occlusal tooth wear, and is not constant over time [5, 14]. Only some individuals were affected by increased wear, primarily related to parafunctional habits (such as bruxism) and/or dental erosion [5, 14, 17, 25]. Although tooth wear is much less in the urban population it is also subject to much greater variability compared to age (Table 2). This contributes to the unreliability of using tooth wear to assess age in urban subjects [1, 17, 21, 26, 27].

There is no straightforward comparison between our data and data obtained from other studies. Most studies do not use dental wear as an isolated indicator [6, 8, 9, 21]. Furthermore there are several techniques for determining the amount of wear of tooth structure [1–3, 6–8, 13–15, 17, 21, 22]. Some methods require the extraction of teeth for histology [1, 13, 21] which can be expensive to carry out, and do not apply to live assessments. Other methodologies use radiographs and CT scans [1, 13, 21]. These too are costly and potentially invasive due to the radiation involved. Such techniques are also inappropriate due to the cultural issues and understanding of the indigenous peoples involved in a study such as this one.

Previous studies of tooth wear have used clinical scoring systems [15, 16, 23, 24] similar to this study. There is no universally accepted scoring system in the literature [10, 14] and as a result the use of different clinical indices prevents the direct comparison of different populations [14]. Generally these clinical indices are quick, simple, and convenient with good reliability since they usually have well-defined criteria with careful calibration amongst examiners [1, 3, 6]. For the Xingu indigenous, which are living in a semi-isolated environment, a requirement to move to an urban center to perform an invasive method would result in a high resistance to participation in the study. In addition, the ethical issues involved in such an approach are considerable (including informed consent and radiation exposure).

Previous studies using tooth wear as an isolated indicator or in combination with others to determine chronological age [1, 7, 10, 11] have not obtained correlation as high as those found in this study of indigenous populations.

Currently most indigenous villages have birth records; however, there are still a large number of unregistered people whose ages are unknown. It is of great importance to establish simple and reliable ways to predict chronological age. The high correlation found in our study between age and tooth wear demonstrates that the recording of tooth wear can be used to help in determining the age of unregistered Amazon indigenous people.

Chronological age estimation of a given individual should be performed using indicators developed for the population to which the individual belongs [6, 9, 14, 21]. Obviously the margin of error is smaller when there is a greater similarity between the individual being examined and the subjects of the sample used for establishing the benchmark [6, 9]. To follow such an approach would require the compilation of regional tables which is not always possible. The ideal method for assessing age is one which achieves the best estimate by basing it on all of the available relevant data [1, 6, 10, 21].

The main goal of this research is to provide a straightforward and efficient method to assist in age determination of newly contacted indigenous tribes. In this case, since tooth wear depends on so many factors, a newly contacted indigenous tribe could present with more or less tooth wear in comparison to the groups analyzed in this present study. Consideration would be given to relevant causative factors such as differing dietary habits, higher or lower amounts of abrasive particles in food depending on the region where they forage, differences in para-masticatory activities and other factors affecting tooth wear. A single model based on examinations in all three populations is proposed as a global model for indigenous tribes (Fig. 3), since the data consistently showed that tooth wear is strongly associated with chronological age in these Amazon indigenous populations. However, as the 95% prediction intervals for aging individuals are quite wide, we caution that dental wear age estimates should not be used as the only means to age indigenous individuals.

## Conclusion

Similar to previous reported studies, tooth wear appears to be a poor estimator of chronological age in the urban population used as a control group, however, it has a strong relationship with chronological age for remote Amazon indigenous populations, as represented by the sample in this study. Analysis of tooth wear can be used as a simple and efficition method to assist in the determination of the chronological age of newly discovered and contacted indigenous populations within the Amazon region. Such an approach would also be likely to be useful in other regions of the world where recently discovered indigenous populations are found.
